# Molecular trafficking between bacteria determines the shape of gut microbial community

**DOI:** 10.1080/19490976.2021.1959841

**Published:** 2021-08-30

**Authors:** Seenivasan Boopathi, Danrui Liu, Ai-Qun Jia

**Affiliations:** School of Life and Pharmaceutical Sciences, Key Laboratory of Tropical Biological Resources of Ministry Education, State Key Laboratory of Marine Resource Utilization in South China Sea, Hainan University, Haikou, China

**Keywords:** Microbiota, contact-dependent interaction, nanotubes, quorum sensing, social behavior

## Abstract

Complex inter-bacterial interactions largely influence the structure and function of the gut microbial community. Though several host-associated phenomena have often been shown to be involved in the stability, structure, and function of the gut microbial community, the implication of contact-dependent and contact-independent inter-bacterial interactions has been overlooked. Such interactions are tightly governed at multiple layers through several extracellular organelles, including contact-dependent inhibition (CDI), nanotubes, type VI secretion system (T6SS), and membrane vesicles (MVs). Recent advancements in molecular techniques have revealed that such extracellular organelles function beyond exhibiting competitive behavior and are also involved in manifesting cooperative behaviors. Cooperation between bacteria occurs through the sharing of several beneficial molecules including nucleic acids, proteins, metabolites, and nutrients among the members of the community, while competition occurs by means of multiple toxins. Intrinsic coordination between contact-dependent and contact-independent mechanisms collectively provides a fitness advantage and increased colonization resistance to the gut microbiota, where molecular trafficking plays a key role. This review is intended to provide a comprehensive view of the salient features of the different bacterial interactions and to highlight how microbiota deploy multifaceted organelles, for exerting both cooperative and competitive behaviors. We discuss the current knowledge of bacterial molecular trafficking and its impact on shaping the gut microbial community.

## Introduction

The gut consists of a dynamic environment that accommodates polymicrobial communities. Bacteria within the polymicrobial communities change their behavior in response to the fluctuating signals and metabolites.^1^ The shaping of the composition of the human gut microbiome is critically governed by several elements including host genetics, diet, and environmental factors,^2–5^ whereas the implications of inter-bacterial interactions have been largely underestimated. Interactions between bacteria are a prerequisite for the trading of QS traits or for antagonistic molecules to maintain the healthy physiology of the host, thereby mitigating the effect of the pathogens.

Intrinsic interactions between microbiota enable them to display synchronized group behavior to produce several molecules, including polysaccharide-utilizing enzymes, siderophores, toxins, biofilm, and other QS traits. Such public goods are meant to be shared among the members of the community, which provides fitness to the participating members of the community. Additionally, genome plasticity also critically determines the physiology of the microbiota. The gut microbiome evolves in response to the changing environment by acquiring foreign DNA, which either increases the fitness of the microbiota through the adaptation of novel metabolic genes or escalate detrimental effects by disseminating antibiotic resistance genes to pathogens.^6^ Genes that are present on mobile genetic elements (MGEs) could confer adaptive attributes to the microbiome, such as antibiotic resistance, detoxification of bile salt, degradation of mucus, biosynthesis of capsular polysaccharides, utilization of polysaccharides, and sporulation.^6,7^ According to the Black Queen hypothesis, bacteria usually undergo genome reduction when they acquire essential nutrients from the surrounding environment, which may be supplied by the other members of the community.^8^ Conceivably, there has been a correlation between nutrient availability and bacterial interaction, wherein abundant nutrient concentrations lead to negative interactions between microbes.^9^ In support of this, sequence analysis has revealed that gut bacteria lack at least one metabolic pathway in their genome, due to which 64% of the tested gut bacteria have been found to be auxotrophs, leading them to depend on prolific external resources for their survival and growth.^10,11^ Therefore, the acquisition or loss of a specific biosynthetic pathway in bacteria has been found to cause metabolic dependency on the surrounding environment,^12,13^ which is likely to implicate obligate cross-feeding mechanisms. It is therefore tempting to speculate that the functionality of the microbiome tends to change with respect to the acquisition of genetic material/loss of essential genes. Consequently, genotypic heterogeneity emerges within the same species, which results in metabolic interdependencies.

The gut is attributed to have peristalsis with a fluid flow. Bacteria residing in such a dynamic environment experience more fluid flow than those at the center of the community, leading to heterogeneity in the QS.^14^ Since QS signals get diffused in the fluid flow, bacteria at the outer edge of the community need to utilize an alternate mechanism for successfully initiating QS. Further, owing to the existence of such diverse and heterogeneous chemical environments, cells within the spatially organized bacterial population are unlikely to have equal access to their essential nutrients,^15^ which forces them to depend on each other for nutrients. Therefore, heterogeneity among the clonal population has necessitated inter-bacterial interactions for the sharing of multiple essential commodities.

To survive in such metabolically heterogenic environments, bacteria adopt various mechanisms to interact with neighboring cells. These strategies have been broadly classified into two categories *viz*., contact-dependent and contact-independent. Major secretory machineries of the bacteria, which include CDI, T6SS, T7SS, nanotubes, and MVs, have been previously considered as weapons that could merely translocate toxins into the target cells. However, with the advent of recent molecular techniques, our current knowledge has improved to elucidate the cooperative behaviors of such machineries, affirming that they are being utilized by bacteria for exerting dual behaviors. To the best of our knowledge, until now, no comprehensive review has been published on the multiple secretory machineries of bacteria. Specifically, the possible existence of such machineries in human gut-associated microbes for exerting both cooperative and antagonistic behaviors. In the present review, we aimed at collating current knowledge of complex inter-bacterial interactions that are likely to occur in the gut-associated microbial community through multiple machineries. We also provide evidence for the impact of such secretory machineries on shaping the gut microbial community.

### Contact-dependent interactions

The contact-dependent interactions are very essential and are considered as primary means of successful community existence and functions. The exchange of essential metabolites between cells is likely to occur when the group of cells persists as aggregates.^15^ Cell-to-cell interactions thus become an imperative attribute to coordinate metabolism and division of labor. However, cell-to-cell interactions and kin recognition are essential for the coordination of multicellular function, for the microbiota to co-localize their potential and suitable partners for establishing stable and continuous interaction over a period of time. Spatial organization of the gut microbiota is important for establishing physical interaction that determines the function of the community.^16^ The following sections essentially discuss the different types of contact-dependent interactions reported in bacteria.

### CDI-mediated interaction

Contact-dependent inhibition (CDI) is considered as a well-known example of a subset of type V secretion system (T5SS).^17^ Bacteria utilize the CDI system to translocate the toxin domain of CdiA into the neighboring cells, once cognate receptors are recognized on the surface of the target cells.^18^ Self-bacteria are successful in neutralizing the effector proteins with the aid of immunity proteins, whereas non-self types are susceptible to the toxins. Bacteria utilize such CDI system for exerting both inhibitory activity and kin recognition. It was believed that CDI-mediated interaction occurs between closely related bacteria, whereas recent report suggests that cross-species effector delivery also occurs due to the promiscuous nature of the class II CdiA receptor-binding domain.^19^ They found that *Enterobacter cloacae* deliver effectors into diverse *Enterobacteriaceae* species such as *Escherichia, Klebsiella, Enterobacter*, and *Salmonella* spp. Thereby, CDI shapes the community composition of *Enterobacteria* spp. in the niche, suggesting that CDI mediates cross-species interaction for kin recognition and competition, and might thereby determine the structure and function of the local community.

**Figure 1.**
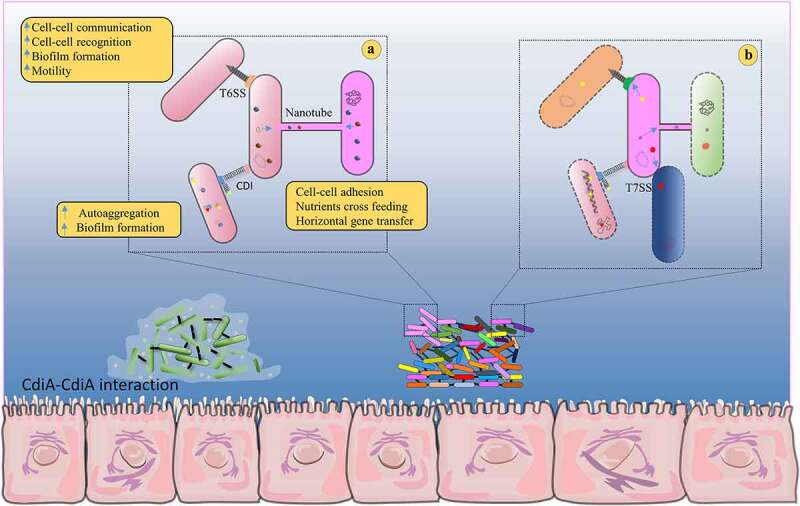
Different set of contact-dependent mechanisms of enteric bacteria. (a) Schematic diagram represents how bacteria interact with cooperatives in a contact-dependent manner, where CDI, T6SS and nanotubes provide fitness advantage to bacteria by facilitating cooperative behaviors.^19–22^ Each mechanism plays an imperative role in bacterial survival. (b) Inhibitor cells kill target cells by translocating toxins through CDI, T6SS, nanotubes and T7SS.^19,23–25^ CDI and T6SS mediate interaction between gram-negative bacteria, whereas T7SS mediates interaction between gram-positive bacteria

Multiple studies in this domain demonstrated that the CDI system is also found to be involved in social behaviors, which are distinct from their regular competitive behavior (Figure 1a). A positive correlation between QS and social behaviors has been reported in several bacteria, suggesting that CDI is controlled by the QS mechanism (Supplementary Figure 1). The series of signaling systems are appeared to be activated upon receiving effector proteins in the recipient cells, which in turn induces biofilm formation and phenotypic changes, thus holistically exerting community behavior.^26^ Such CDI mediated transcriptional changes have been termed as contact-dependent signaling (CDS). It was perceived that CDS might function as a fine-tuning mechanism, playing a pivotal role in structuring the bacterial community.^26^

Interestingly, *cdi* loci are present on the genomic island, revealing that *cdi* toxin/immune system is likely to be transferred between bacteria through HGT.^18^ This was confirmed by the presence of the CDI system in probiotic *Escherichia coli* (Nissle 1917).^27^ It could be conceived that the CDI system has been the characteristic attribute of not only pathogens but also of gut microbiota. As a consequence of HGT, bacterial genome have frequently been found to consist of many orphan *cdiA/cdiI* modules at the downstream of the CDI system,^28^ denoting that CDI system provides an additional fitness advantage to the harboring cells. The presence of multiple *cdi* loci in a cell corroborates that they work together synergistically and exhibit strong activity against competitors even at the low expression of CdiA.^29^ The CDI system was found to be different among cells and within species due to the polymorphic nature, which preferentially selects members of the local community.^20^ As a result of HGT, *cdi* genes are widely present in genetically unrelated species.^28,30^ The CDI system was found to be involved in aggregation, where the adhesion of CdiA–CdiA exoprotein allows the aggregation of CDI^+^ harboring cells.^31^ Thus, such receptor-independent auto-aggregation co-localizes genetically diversified bacteria, where *cdi* genes-acquired cells score over other CDI^‒^ cells. It could be conceived that CDI-mediates both inter- and intra-species interaction. However, CDI-mediated inter-kingdom interaction has not yet been studied. The cooperative phenotypes effectively communicate with each other if they are present in an aggregated form in the niche. Therefore, it is postulated that CDI also helps in QS by assorting the CDI^+^ cells at a niche, where secreted QS signaling molecules could reach the intended bacteria to activate QS. The CDI-mediated biofilm induction denotes the intrinsic interconnection between the CDI and QS. CDI-mediated competition is effective when CDI^+^ cells are present in the niche at high cell density. This was in turn found to cause increased cell–cell interaction and a swift inhibitory effect on target cells, thereby restricting their expansion and influencing the composition and spatial arrangement of the bacterial community.^17^ Therefore, co-localization and cell-to-cell contact between bacteria are crucial factors in determining the physiology and ecology of the microbial communities.

CDI could play an essential role in governing community composition and function through two mechanisms: (i) colocalization of high-density CDI^+^ cells in the niche by means of CdiA–CdiA interaction and CdiA-receptor interaction, which subsequently facilitates the delivery of effectors to the competitors of the niche, thereby promoting initial colonization.^19,31^ (ii) Protecting microcolonies from invading competitors through inter-species toxin delivery, for which bacteria utilize class II CDI system.^19^ By using several bacteria as model organisms, it was found that the CDI system is essential for structuring the community.^32,33^ It is evidently proved that bacteria, which are defective in the CDI system, are unable to architect biofilm.^31,33^ Thus, the CDI system governs spatial arrangement, composition of the community, as well as the behavior of bacteria, which includes cell–cell aggregation, biofilm formation, and modulation of transcriptomes in the recipient cells, besides stress tolerance. Therefore, the CDI system might be an unexplored phenomenon in gut microbiota, which provides fitness to CDI^+^ cells by regulating both cooperative and competitive behaviors. Though CDI-mediated interaction has been demonstrated in governing the composition of the local community, the impact on the entire gut-microbial community in an *in vivo* condition has not yet been explored. Therefore, understanding the impact of CDI in manipulating the structure and function of the gut microbial community will broaden our knowledge toward developing novel strategies.

### T7SS-mediated interactions

Contact-dependent mechanisms have also been found in host-associated gram-positive bacteria. Gram-positive phylum Firmicutes possess T7SS, which is functionally equivalent to T6SS of gram-negative bacteria.^34^ T7SS utilizes effectors-immunity (EI) repertoires that usually occur with large variations within the same species, revealing their role in kin recognition and competition.^34^ Comparative genomic analysis of *Streptococcus* spp. revealed that T7SS encoding machinery is located on genomic islands,^35^ suggesting that T7SS might exist in different species of gram-positive bacteria, allowing cross-species interaction.

Genes belonging to the LXG protein family are abundantly present in different species of Clostridiales, Bacillales, and Lactobacillales, in which most of the gut microbiota are classified.^23^ The LXG proteins-mediated antagonism effectively defines the composition of the community that is rich in Firmicutes. Commensal *Streptococcus intermedius* produces three different LXG family of polymorphic toxins such as TelA, TelB, and TelC, along with their respective immunity proteins TipA, TipB, and TipC to neutralize self-intoxication. Toxin-antitoxin systems thus provide a fitness advantage to the producer organisms. Wild-type cells have been found to utilize T7SS to intoxicate TelC-susceptible cells in a contact-dependent manner.^23^ T7SS-mediated antagonistic effect has been observed between closely related gram-positive organisms such as *S. intermedius* (defective in producing immunity proteins such as *tipA* and *tipB), S. pyogenes*, and *E. faecalis*, whereas such activity has not been shown significantly against gram-negative species belonging to the proteobacteria and bacteroidetes phylum.^23^ Thus, T7SS mediates inter- and intra-species interaction, suggesting their role in determining the composition of gram-positive bacteria in the community. Due to its versatile nature, T7SS also mediates interaction with host cells for the exchange of virulence factors.^36^ Numerous immunity proteins are accumulated in a taxonomically distinct group of microbiota, suggesting that microbiota increase their survival fitness by avoiding intoxication using polyimmunity loci or polyimmunity proteins.^37^ Thus, diverse toxin-antitoxin modules, acquired through HGT, serve as a common reservoir for several secretory systems utilized by bacteria for the selection of members in the community.^38^ For instance, the MuF toxin family has been found in the temperate phages of Firmicutes.^39^ The mining of toxin diversity in gut microbiome and identifying their unknown biochemical function could help us to develop a novel strategy to curb pathogens. However, the global impact of T7SS machinery on the structure and function of gut microbiota has not been studied so far. Unraveling the link between gut microbiota and T7SS will provide novel insights that could spur our research in a new dimension.

### Nanotubes-mediated interactions

Recently, a novel type of bacterial communication has been discovered, in which bacteria establish physical contact with the neighboring cells through conduits.^40^ Such tubes serve as intercellular conduits for the exchange of various cytoplasmic molecules, thereby displaying co-operative or antagonistic behaviors toward neighboring bacteria.^21,24,40^ Certain biomolecules are unable to cross the bacterial membrane due to their unique biochemical characteristics.^15^ In such cases, nanotubes might serve as a channel for the transportation of chemically diverse molecules between cells. The membrane integrated proteins of the CORE complex, components of type III secretion system (T3SS), were found to serve as a platform for the assembly of both nanotubes as well as flagella in gram-positive bacteria.^41^ Interestingly, the orthologue of CORE complex has been involved in injectisome formation in gram-negative bacteria.^42^ Since CORE complex is functionally conserved among different bacterial species, nanotubes-like structure were reported in diverse commensal guts.^43–46^ Not surprisingly, several enteric pathogens were also reported to produce nanotubes-like structure,^47–50^ denoting that nanotube formation could be an inherent nature of many bacteria. It has been frequently observed that microbiota eliminate enteropathogens from the niche in a contact-dependent manner. For instance, contact-dependent interaction between *Salmonella enterica* subsp. *enterica* serovar Typhimurium and fecal bacteria has led to the loss of viability of *S*. Typhimurium.^51^ Similarly, *Lactococcus piscium* was found to inhibit the growth of *Listeria monocytogenes* in a contact-dependent fashion.^52,53^ Perhaps commensals kill those pathogens by translocating toxic molecules through the nanotube network. However, the stability and functions of nanotubes for active molecular trading *in vivo* conditions needs to be further elucidated. Apart from molecular trading, nanotubes are perceived as an anchoring factor required for cell-surface and cell–cell attachment.^54^

In nutrient-deprived conditions, nanotube formation is induced among bacteria to facilitate the movement of nutrients to the intended bacteria.^21^ Moreover, auxotrophs acquire essential amino acids from the donor cells through nanotubes, which results in the restriction of feedback inhibition of the respective amino acid biosynthetic pathway. Thus, auxotrophs enable the overproduction of amino acids in donor cells.^55^ Therefore, the network of nanotubes provides a selective fitness advantage to bacteria to adapt to the environment.^56^ In line with other sensing mechanisms, nanotubes are also likely to be involved in discriminating self and non-self cells through EI repertoires (Figure 1b).^24^ In a natural environment, bacterial fitness was found to be increased, by not investing in the cost of their survival; instead, they hijack costly products derived from the neighboring cells. Thus, noncooperative bacteria get access to such public goods, though their contribution is none.^15^ To overcome such problems, nanotubes might be engaged in privatizing goods by restricting access to the other bacteria, in which producers securely deliver their public goods to the intended partners through nanotubes. Bacteria were found to efficiently communicate with far distantly located bacteria through the formation of elongated nanotubes,^21,57^ suggesting that bacteria could exchange cargo to the outer edge of the microcolonies.

Since nanotubes have been implicated in inter-bacterial interaction, the question rises as to whether the QS mechanism can control nanotube formation. However, we are still at the much early stage to predict the link between nanotubes and QS. However, even a single cell can produce elongated nanotubes on a solid substratum, as opposed to the QS principle, in which multiple cells are required to determine the production of social traits. Nanotubes might have been another form of embodiment of MVs, which have similar membrane structure to MVs. Bacteria utilize cell wall remodeling enzyme LytC and its activator LytB for the extrusion of nanotubes from the donor cells and penetration into the recipient cells.^58^ LytB localizes on the growing nanotubes and reaches recipient cells for the activation of LytC for the successful penetration of nanotubes into the recipient. LytB of the donor cells can activate the LytC of different species of recipients for establishing a nanotube network. However, the compatibility between LytB and LytC, which originate from different cells, determines intra- and inter-species interaction in a multi-species community. It is tempting to speculate that bacteria utilize LytB that functions as a signal to target a suitable partner for establishing a nanotube network in the complex community for exerting cooperative or antagonistic behaviors. In addition to LytB and LytC, other proteins such as LytE, LytF and SigD are also involved in nanotube formation.^59^ Thus, the cell wall hydrolases and CORE complex system determine inter-bacterial interaction.

Though several reports have highlighted the impact of nanotubes between two individual bacteria, their impact on the global bacterial community has not been explored yet. However, based on the nature of cargo materials, we propose that bacteria might utilize nanotubes for multiple functions that could potentially govern the community composition. Nanotubes are known to transfer genetic material, nutrients, and other essential commodities among the members of the community. The network of nanotubes between bacteria appears as a syncytium-like multicellular consortium, in which essential metabolites, including QS signals, are likely to be transported to the intended members of the community. Therefore, such nanotube networks could be viewed as a platform for the repair mechanism, where nanotubes could ensure the function of the community by facilitating proper QS regulation even in the outer edge of the community that is usually prone to metabolic heterogeneity due to the fluctuating environment. Therefore, it is tempting to speculate that nanotubes might govern the functional diversity of the microbial community. Since the discovery of nanotubes, only a few research groups are actively working to decipher their functional aspects. However, their novel findings are derived from one-to-one bacterial interaction experiments. Deciphering the role of nanotubes in a multi-species community will update our current knowledge of bacterial interaction, which could be further utilized to develop new therapeutic interventions.

### T6SS-mediated interaction

T6SS is a complex and well-characterized nano-machinery in gram-negative bacteria, which was found to intoxicate target cells in a contact-dependent manner. It was believed that T6SS is restricted to proteobacteria, which are minor members of the gut microbial community. Recently, T6SS was also found rich in the order of *Bacteroidales*.^60^ T6SS has been classified into three different genetic architectures (GAs), in which GA1 and GA2 are often seen on the conserved integrative conjugative elements and disseminated among different species of gut *Bacteroidales*.^25,60^ Metagenomic analysis of human gut samples revealed that GA3 has been found specifically in *Bacteroides fragilis*.^61^ They also found that GA3 containing *B. fragilis* is more common in the gut microbiome of infants than in adult gut samples, suggesting that T6SS critically governs the gut microbiome composition at an early stage of life. The protein composition of T6SS of proteobacteria differs from that of Bacteroidetes,^62^ suggesting that pathogens and commensals could be relatively discriminated based on architectural proteins.

Genome analysis of *Bacteroidales* revealed that GA1 and GA3 loci have been found in the same genome of the bacteria, whereas GA2 T6SSs have not been observed in the genome along with either GA1 or GA3.^60,63^ It is therefore conceivable that the presence of multiple T6SS loci in the genome could confer strong protection against invaders. GAs of T6SS loci of gut microbiota have been found to contain variable regions that encode diverse toxins, which appear to be functionally different from the known repertoires.^63^ The distribution of T6SS EI pair in human gut microbiome samples encourages the notion that this pathway plays a large role in defining the members of the community through competition and selection. Due to its versatile nature, T6SS has been found to mediate microbe–microbe interaction in an intra- and inter-species manner, as well as host–microbe interaction.^25,64^ *B. fragilis* has been known to utilize T6SS to discriminate closely related organisms.^25^ For instance, symbiotic non-toxigenic *Bacteroides fragilis* has been reported to exhibit colonization resistance against enterotoxigenic *B. fragilis* through strain-specific competition using GA3 T6SS, thus protecting the host from the disease colitis.^65^ It could therefore be postulated that T6SS-mediated interaction confers protection by eliminating local competitors and precisely manipulating the community composition. T6SS has been found to exhibit a profound impact on the gut microbial community, in which GA3 T6SS mediated firing of effectors exceeds 10^9^ times min^−1^ gram^−1^ of colonic content.^66^ It is, therefore, speculated that GA3 can precisely manipulate the composition of the *Bacteroidales* in the community. However, the role of GA1 and GA2 in determining the structure and function of the community is not yet understood clearly.

Recent study using metagenomic data suggests that the members of the bacterial community differ between the samples that contain T6SS+ cells and T6SS**‒** cells. GA3 type of T6SS favors the abundance of Bacteroides in the niche. The abundance of *Bacteroides* is closely associated with the presence of T6SS+ cells, whereas *Oscillospir*a, *Faecalibacterium*, and *Ruminococcus* from the Firmicutes phylum are negatively associated with T6SS+ cells.^61^ Thus, T6SS is critically involved in determining the composition of the gut bacterial community. T6SS also delivers toxins in a contact-independent manner,^67^ which allows them to exhibit antagonistic activity against a broad range of bacteria. This implies that irrespective of kin and non-kin cells, T6SS can deliver effector proteins to all the nearby cells. Kin cells produce immunity proteins that neutralize cognate toxins. Thus, T6SS increases the fitness of the producer cell through competition with other bacteria for niche and nutrients. Thus, T6SS precisely manipulates the composition of *Bacteroidales* through antagonistic activity and kin selection.

It has been well documented in the literature that certain *Bacteroidales* members harbor T6SS encoding machinery along with acquired inter-bacterial defence (AID) gene clusters that provide immunity against different T6SS effector proteins in inter-species and intra-species manners.^68^ Orphan immunity genes that confer protection against T6SS-mediated antagonism are widely present in the human gut microbiome, probably as a result of HGT. Nevertheless, surprisingly, more than 50% of the searched *Bacteroidales* genomes are known to possess recombinase associated AID, which provides ecological fitness to the immunity genes harboring cells. Therefore, the acquisition and preservation of an orphan immunity system in the genome of the *Bacteroidales* is a common mechanism to inhibit competing bacteria.^68^

Surprisingly, T6SS is also involved in exhibiting social behaviors. Bacteria are capable of perceiving T6SS-mediated attacks from neighboring cells, by which they might determine the surrounding population to exert social behaviors.^22^ *Proteus mirabilis* is a low abundant gut microbiota in some human beings, which can swarm outward and exhibit a visible boundary when they meet non-self cells. However, swarms of the same group of cells merge on the solid medium.^69^ Such cell–cell recognition is typically governed by *ids* and *idr* gene clusters, which encode self-identity proteins and *rhs-*related products, respectively.^70^ It was found that proteins such as IdsD and IdE function as strain-specific self-identity determinants.^69^ Interestingly, it may be noted that T6SS is involved in such cell–cell recognition by exporting self-identity determinants from one cell to another cell.^70^ The recognition of self-cells occurs when IdsD from the producer cells interact with IdsE of the sibling cells. Thus, the binding of such nonresident IdsD with resident IdsE leads to the merging of the population.^71^ In contrast, nonresident IdsD remain unbound in non-self cells, resulting in the shift of their lifestyle of incompatibility to co-exist with self-cells. Consequently, non-self cells transiently become tolerant to antibiotics and also display differential gene expression that keeps away those non-self-cells from participating in the production of social traits.^72^ The metagenome of the human gut microbiome has revealed that several variants of IdrD-like genes exist at the lower level, suggesting that IdrD could be the hallmark of low abundant bacteria to survive in a competitive environment.^73^ The display of self-identity determinants provides competitive advantages to the cells due to cell–cell interaction that collectively provides strength in yielding coordination across the clonal population for exerting territorial behavior.^70^

Securing public goods from cheater cells through T6SS is one of the cooperative behaviors of bacteria. The T6SS-mediated killing of non-self competitors creates space between the co-operators and cheaters, thereby restricting cheaters from accessing public goods.^74^ *In silico* analysis has revealed that the genomes of proteobacteria and Bacteroidetes harbor genes for public goods production, which is positively correlated with the increasing number of T6SS.^74^ Notably, secretion of essential proteins and metabolites through the T6SS machinery possibly allows access to other clonemates, regardless of their contribution in the production of those proteins.^75–77^ Thus, sharing of public goods among the members of the community also facilitates cooperation. The T6SS-mediated biofilm formation and motility have also been reported in different sets of bacteria.^78,79^ Thus, the involvement of T6SS has been confirmed in social behavior through cell–cell communication, biofilm formation, securing public goods from cheater cells, nutrient acquisition, and HGT. Since T6SS was prevalently found in the order of *Bacteroidales*, such group behaviors could also exist in gut commensal bacteria. Therefore, studying T6SS-mediated cooperation among gut microbiota will delineate its physiological role in the complex microbial community. Further, identification of the factors required for T6SS-mediated inter-bacterial interaction will provide an opportunity to develop novel tool kits to control pathogen expansion. Since T6SS manipulate the composition of *Bacteroidales*, which is the most abundant gut microbial community, utilization of T6SS for a therapeutic purpose could be a venture with future potential. Altogether, contact-dependent interactions have unique attributes (Table 1) that are exhibited by bacteria to thrive in hostile environments, like gut.

**Table 1.** Different types of contact-dependent interactions and their role in providing fitness advantage to the harboring bacteria

Features of machinery	CDI	Nanotubes	T6SS
Size of the extracellular appendage	CdiA filament extend up to ~33 nm.^80^	Extend up to a few µM from the cell surface.^40,57^	Cell width determines the length of the tail.^81^
Occurrence	Present in α-, β- and γ-proteobacteria, including commensal gut microbiota.^30^	CORE complex is highly conserved in both gram-positive and gram-negative bacterial species. Therefore, nanotube machinery might present across the bacterial kingdom.^41^	Present in Proteobacteria and Bacteroidetes.^60,63^
Components required for assembly	TpsB family transporter and TpsA family exoproteins are required.^82^	Genes such as *fliO, fliP, fliQ, fliR, flhB*, and *flhA* are known as CORE complex encoding proteins serve as a platform of nanotube assembly.^41,42^ *lytE* and *lytF* have also been found to be involved in nanotubes formation, where *sigD* functions as an important regulator.^59^	Genes such as *tssA, tssB, tssC, tssD/hcp, tssE, tssF, tssG, tssH/ClpV, tssI/VgrG, tssJ/SciN, tssK, tssL, tssM* are required for its assembly in proteobacteria.^83^ However, conserved T6SS components of proteobacteria such as TssA, TssJ, TssL and TssM have not been found in the genome of gut *Bacteroidales*.^60^ T6SS of *Bacteroidales* harbor unique proteins, including TssN, TssO, TssP, TssQ and TssR which might compensate for the function of the missing proteins.^60,62^
Exchange of essential nutrients	Well known for toxin exchange only.^31,38^	Facilitate the reciprocal exchange of essential nutrients.^21,24^	Secrete micronutrients scavenging molecules.^75–77,84^
Directionality	Deliver effector proteins from donor to recipient.^1,85^	Nanotubes can exchange molecules bidirectionally. Bacteria kill target cells by translocating WapA toxin via nanotubes; in return, they extract essential nutrients from the prey cells concurrently.^24^	Deliver effector protein from donor to recipient. Reciprocally, extract genomic DNA from the recipient through T6SS machinery.^86^
Cargo molecules	Due to the polymorphic nature, CdiA-CT domain exerts various distinct toxicity, including membrane ionophore toxin, tRNase, rRNase, and DNase. Such toxic molecules are translocated into the recipient cells.^85,87–90^	Trading of various molecules including DNA, proteins, toxins, amino acids has been reported.^21,24,40^	Exchange effector proteins and genetic material.^91–93^
Target organism for interaction	Class I CdiA shows species-specific activity, whereas class II CdiA exhibits a broad range of activity.^19^	Nanotubes mediated interaction was found between both gram-positive bacteria and gram-negative bacteria and even between genetically unrelated organisms, as well as with mammalian cells.^40,42^	T6SS effector proteins exhibit strong antagonistic activity against different species of Bacteroidales, but not toward strains belong to proteobacteria.^25^
Receptor specificity for Cell–cell interaction	Class II CdiA specifically binds with OmpC and OmpF proteins of recipient cells, whereas Class I and Class III CdiA bind with BamA and Tsx of recipient cells respectively.^94–96^	Cell wall remodeling enzyme LytC of recipient cell and its activator LytB of donor cells are involved in cell–cell interaction. Interspecies compatibility between LytB of the donor cells and LytC of recipient cell determines interspecies interaction.^58^	Specific receptor proteins not yet discovered for inter-bacterial interaction. T6SS also interacts with neighboring bacteria in a contact-independent manner.^67^
Cell–cell interaction in dynamic condition	CDI-mediated killing was observed between bacteria that grow in shaking liquid culture.^97^	Even in a dynamic environment, bacteria establish a connection between cells through nanotubes.^21^	Successful cell–cell interaction in the gut environment is reported using animal model.^61,66^
Impact on biofilm formation	Aggregation of bacteria through CdiA-CdiA interaction or Cdi-receptor protein interaction appears to be involved in biofilm formation.^31^	Nanotubes-mediated cell–cell interaction co-localizes all cells at a given space which could probably induce biofilm formation	T6SS mediated biofilm formation has been reported in several bacteria.^78,79^
Role of Quorum sensing	QS-mediated *cdi* gene expression has been found in *Burkholderia thailandensis*.^98^	Bacteria could efficiently connect far distantly located bacteria through elongated nanotubes (more than 50 µM length).^21,57^ Co-localization of group of cells through nanotubes network might facilitate cell-cell communication	QS regulates T6SS-mediated interaction.^99^
Time required for interaction	After one hour, translocation of effector protein from donor cell to recipient is observed.^90^	Bacteria produce ~57 µm length nanotube after 70 min.^55^ Therefore, nanotube network establishes connection between cells within short duration.	Lysis of target cells is observed after two hours.^100^
Dependency on cell-to-cell contact for the delivery of molecules	Translocation of effectors occurs upon contacting recipient cells.^26^	Contact-dependent interaction is well established. But the function of nanotubes without contacting recipient cells is not yet reported.^24,40^	Though effector proteins are exchanged through contact-dependent interaction, in some cases, T6SS exports proteins in the extracellular milieu.^67,75^

### Contact-independent interactions

The QS mechanism has been a well-known and well-documented example of contact-independent interaction. Such mechanism modulates global gene expression in the broad members of the community, which could be conceived as an advantage over contact-dependent interactions. Bacteria produce numerous QS traits through the QS mechanism, which are found to help them to display either co-operative or antagonistic behavior for their survival. Effective communication between bacteria could be achieved when the cells are in close proximity, whereas interactions with distal cells have primarily relied on the flow in a dynamic environment such as gut. In response to the fluctuating fluid flow, bacterial QS regulation has often been found to adopt an ON/OFF mode.^14^ Besides, degradation of the QS signals due to biotic and abiotic factors also inhibits bacterial communication.^101^ Another drawback of this mechanism is that certain signals are liable to chemical modification or diffuse in the fluid dynamic environment.^15^ Our current knowledge of contact-independent interaction in governing the structure and function of the gut microbial community is summarized in the following sections.

### Membrane vesicles-mediated interactions

**Figure 2.**
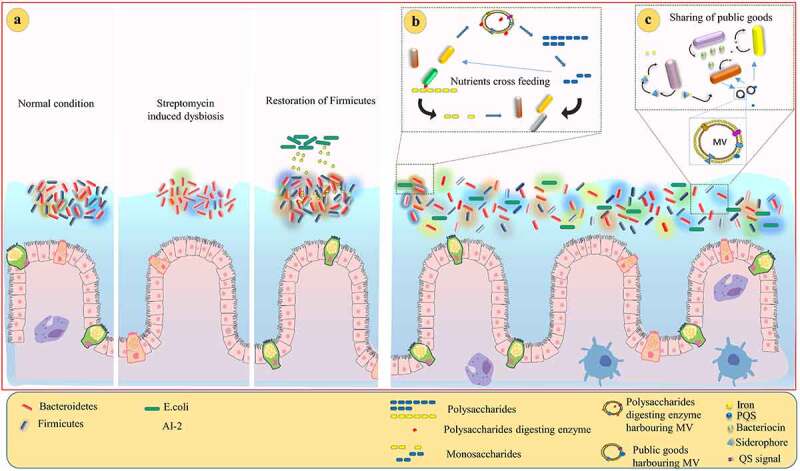
Schematic illustration of how enteric bacteria cooperate with neighboring cells in a contact-independent manner. (a) Streptomycin treatment selectively eliminates Firmicutes population in the gut. QS signal AI-2 restores the balance between Bacteroidetes and Firmicutes after antibiotic induced dysbiosis.^106^ (b) Microbiota secretes polysaccharide-digestive enzymes in the milieu or through membrane vesicles, which digest the polysaccharides into monosaccharides that can be accessible to other members.^107^ (c) Bacteria secrete numerous molecules as a public goods to share with their community members.^84,108^

During complex interactions between bacteria, they secrete several molecules, certain of which are hydrophobic, liable to be inactive in the extracellular environment.^102^ Such a phenomenon necessitates the bacteria to utilize alternative mechanisms that can perform the exchange of such cargo effectively. Notably, MVs mediate intercellular communication through the exchange of different types of biomolecules, including proteins, carbohydrates, and nucleic acids.^103^ Contrary to the earlier belief that only gram-negative bacteria can produce MVs, gram-positive bacteria too have been found to do so.^104,105^ The public goods produced by gut microbiota are accessible to them not only for self-use but also to other members of the community, regardless of their contribution (Figure 2c). Members of Bacteroidetes were found to be more stable in the human gut over time.^109^ This could be attributed to their ability to utilize diverse groups of polysaccharides.^107^ Different species of Bacteroides produce MVs, which carry polysaccharide-digesting enzymes that facilitate cooperation with neighboring cells by serving as a public goods.^107,110–112^ The gut microbiota-derived glycoside hydrolase/polysaccharide lyases harboring MVs have been known to be functionally active even at a distance from the producers, which tend to liberate digested products that can be accessible not only to them but also for the non-producers,^107^ thus increasing the fitness of the cooperative phenotypes by sharing metabolic by-products (Figure 2b). *Bacteroides thetaiotaomicron* defective in utilizing amylopectin or levan could not grow as monoculture, whereas in the presence of wild-type, the mutants have shown growth by utilizing public goods produced from the wild-type.^113^ It was also found that during such intricate interaction among *Bacteroidales*, non-producers did not affect the fitness of such public goods producers adversely; rather, they increased the fitness. However, cells that produce enzymes have more access to utilize polysaccharides than non-producers.^113^ These are the best examples of cooperation between gut bacteria through public goods. Additionally, reciprocal cooperation has also been evidentially proved in the gut environment. Though outer surface glycoside hydrolases are meant for the utilization of inulin digestion, *B. ovatus* (*BO*) directly utilizes imported inulin for its fitness. It is surprising to note that neighboring *Bacteroides vulgatus* (*BV*) utilize digested products of inulin available in the niche due to outer surface glycoside hydrolases of *BO*. Further, reciprocally, *BV* has been found to provide a beneficial effect to *BO* by detoxifying inhibitory molecules or releasing growth-promoting factors.^113^ Cross-feeding of nutrients has been demonstrated between *Bifidobacterium adolescentis* and butyrate-producing anaerobes.^114^ Such reciprocal cross-feeding might be facilitated by MVs. The fitness of the gut microbiota increases when they utilize MVs associated public goods. A large number of Bacteroides species have been found to produce MVs which carry surface-associated cephalosporinase that degrade β-lactam antibiotics in the vicinity. Thereby, MVs protect commensals and pathogens from β-lactam antibiotics.^115^ In response to β-lactam antibiotic imipenem, *Stenotrophomonas maltophilia* secrete β-lactamase containing MVs that degrade β-lactam from the vicinity and confer protection not only to their clonemates but also cohabitants. Thus MVs provide protection in an intra- and inter-species manner.^116,117^ QS signal *Pseudomonas* quinolone signal (PQS) also functions as an iron scavenger. Notably, MVs specifically deliver the cargo to the target cell. Enterobacterium *Buttiauxella agrestis* produces MVs that selectively interact with the same genus.^118^ Likewise, *P. aeruginosa* secretes probable T6SS substrate TseF, that directly binds with PQS-harboring MVs. The interaction of TseF bound MVs with Fe(III)-pyochelin receptor FptA and the porin OprF, facilitates the delivery of iron molecules to specific cells.^84^ It is, therefore, speculated that MVs could be delivered to the intended bacteria in the complex polymicrobial community. As with other secretory machineries, the formation and secretion of MVs is tightly regulated by QS mechanisms.^117,119^ Reciprocally, MVs mediate QS regulation through the delivery of structurally diverse QS signaling molecules, including cyclic PQS and acylated lactone signals,^102,104,120^ which tempted researchers to speculate that MVs could facilitate QS in a dynamic environment like the gut (Supplementary Figure 1). It is difficult to detect cognate QS signals in the complex gut environment due to its fluctuating and dynamic nature. MVs harbor concentrated QS signals which are likely to be delivered to the intended bacteria, as opposed to the classical diffusion-based pathway where QS signals are equivalently distributed in the environment.^104^ Conceivably, a single MV containing concentrated QS signals is sufficient to activate QS regulation in the recipient cell.^104,121^ Thus, MVs-mediated QS regulation may influence population-wide changes. The association between QS and MVs has not been studied so far in the gut microbiota. However, it is a potential opening for manipulating the gut microbial community. We propose that gut microbiota-derived MVs could be involved in determining the community composition through cargo materials, including genomic material and QS molecules. MVs exceptionally contribute to HGT, where DNA-harboring MVs originating from diverse bacterial species can fuse with distantly located cells, whereas in the conjugation process, one- to- one transfer occurs. The gut microbiota, especially *B. fragilis*, has been known to excrete novel antimicrobial protein through MVs.^122^ Different species of lactobacilli have been shown to produce MVs that contain various protein components, including bacteriocin,^105^ suggesting that such MVs are implicated in inter-bacterial competition.

Thus, MVs of microbiota have been involved indispensably in displaying co-operative behaviors by functioning as carriers for QS regulations (i.e., biofilm formation) and nutrients cross-feeding among close relatives. MVs are also implicated in determining the community composition by disseminating toxins to kill competitors, thereby shaping the microbial community structure. Thus, gut microbiota-derived MVs are involved in maintaining the proper health of the host.^123^ Essentially, the identification of MV producers from the complex polymicrobial community is required for the understanding of their functional role. However, the purification of different populations of MVs from the large metabolic pool is still a challenging task. Development of improved protocols for the purification and enrichment of MVs from complex gut microbial samples and simultaneous exploration of microbiome composition can elevate this avenue to the next level.

### Quorum sensing mediated interaction

**Figure 3.**
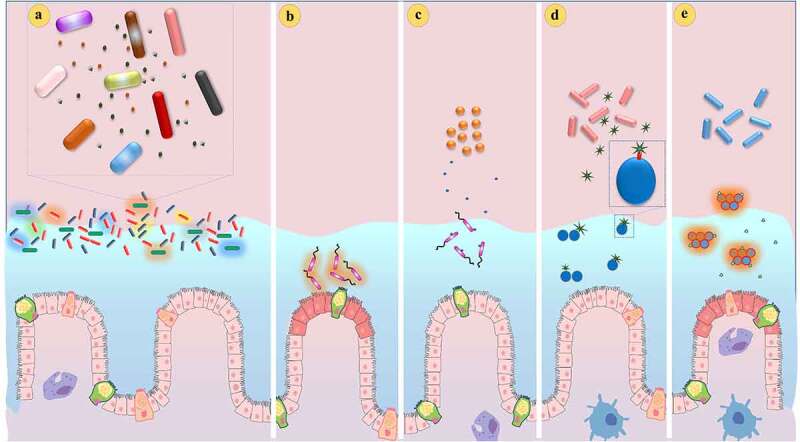
Diagrammatic representation of how microbiota competes with pathogens in a contact-independent manner. (a) Microbiota secretes toxic molecules that affect the growth of the competitors.^105^ (b) Colonization of *V. cholera* in the intestine causes inflammation. (c) *Blautia obeum* produces AI-2 like molecule that suppresses the QS mechanism of *V. cholera*.^126^ Similarly, (d) *B. subtilis* restricts the colonization of *S. aureus* through the production of fengycin that competitively binds with QS receptor AgrC of *S. aureus*.^127^ (e) *B. subtilis* defective to produce fengycin is not able to restrict colonization of *S. aureus.*

Quorum sensing is a process in which bacteria produce, detect and respond to signaling molecules to regulate their gene expression in response to their population density and/or species composition of the surrounding community.^124^ The sequencing data of the human microbiome has revealed that 30% of the small proteins appear to be associated with cell–cell communication, in which 9% of the gut metagenome is found to be transmembrane proteins or secretory proteins.^125^ This finding has confirmed that gut microbiota has a sophisticated system for inter-bacterial communication. The microbiota must communicate with the co-operative phenotypes for producing QS traits (public goods), whereas solitary cells are unable to produce enough public goods. Thus, the presence of co-operative phenotypes at high cell density is the prerequisite for the higher production of public goods. For example, probiotic *Lactobacillus plantarum* exerts higher production of QS-mediated bacteriocin only at high cell density, which could effectively eliminate the competitors from the vicinity^108^ (Figure 3a). However, detecting own cell density in a complex environment has been questioned due to the high fluid dynamic environment, like gut. In addition to QS signaling molecules, other QS traits are also involved in interbacterial signaling. For example, siderophore, hydrogen cyanide and rhamnolipids have been found as important mediators for inter-bacterial interaction.^128–130^ Additionally, cell damage induced by the competitors might serve as a signal for the presence of noncooperative phenotypes. For instance, *Salmonella* Typhimurium increases the expression of biofilm formation, antibiotic tolerance, and virulence in response to the T6SS-mediated attack of the competitors.^131^ It is therefore understood now that bacteria exhibit multiple mechanisms to recognize neighboring cells, which could increase the fitness of the community. Interestingly, cheating behavior is not only the attribute of pathogens but also of the microbiota, which confers protection against pathogens. During inflammation, the gut microbiota has limited access to iron, whereas pathogens acquire iron by producing siderophores. Commensal *B. thetaiotaomicron* has been reported to effectively scavenge irons through xenosiderophores such as enterobactin and salmochelin from pathogens.^132^ Thus, it is evident that gut microbiota exploit the costly public goods of pathogens, which provide a fitness advantage to commensal bacteria for resilience from the disease colitis.

Gram-positive and gram-negative bacteria utilize AHL type of signaling molecules and peptides-type of signals, respectively.^124^ The enzyme that is encoded by luxI gene is responsible for the synthesis of AHL. Once AHL binds to the cognate receptor protein LuxR, the transcription of subsequent genes is activated. Interestingly, LuxR encoding genes have often been found in the genome of bacteria without cognate LuxI coding gene. Such unpaired LuxR homologues are known as LuxR solo or orphans. *In silico* analysis revealed that around 80% of the LuxR sequences occur in the bacterial genome without LuxI pair.^133^ Human-associated bacteria harbor one or more LuxR homologs in their genome.^133^ The presence of multiple LuxR homologs within the same genome provides a fitness advantage to the bacteria because LuxR solos can regulate the gene expression in response to signals that originate from diverse bacteria. Mining of LuxR homologues against the human microbiome project revealed that LuxI/LuxR homologues exist in some proteobacteria, but not detected in Firmicutes.^134^ The presence of such LuxR solos is found in diverse environments, denoting their essential role beyond AHL sensing and host–microbe interactions.^133^ LuxR solos tend to eavesdrop on AHLs and non-AHLs produced by other members of the community, thereby regulating the gene expression of those bacteria to increase the fitness and adaptive attributes of the bacteria to survive in adverse environmental conditions.^133^ Enterohemorrhagic *E. coli* (EHEC) is a normal resident of cattle rumen. LuxR homolog SdiA is essential for EHEC for successful colonization in the gut, where SdiA senses AHLs produced by other bacteria. Thereby, EHEC regulate their own gene expression to adapt to the gut environment.^135^ Likewise, commensal *Enterobacter cloacae* utilize sdiA for sensing the AHLs of other bacteria.^136^ *E. coli* senses interspecies signal indole through SdiA receptor, which results in the decrease of biofilm formation.^137^

The existence of transposases-encoding gene adjacent to the *luxR* sequence suggests the possible occurrence of HGT.^133^ The presence of LuxR homologs in non-proteobacteria could be the result of HGT to eavesdrop signals from other bacteria. The acquisition of LuxR through HGT could be considered an evolutionary strategy of the bacteria to adapt to the fluctuating environment, where the receptor recognizes the prevailing QS signals, regardless of their origin. Due to the presence of orphan LuxRs, bacteria can interact with other bacteria in an intra- and inter-species manner. Thus, LuxR solos mediate crosstalk between genetically unrelated bacteria thereby broadens the communication network for long-term persistence. Thus, HGT and QS could determine the functions of the bacterial community. Though multiple LuxR homologs are found in the genome of bacteria, the functional role of LuxR homolog in determining the structure of the gut microbial community is largely unveiled.

Another important QS signal is autoinducer-2 (AI-2) that has been widely reported in different species of bacteria. It has been found that more than 80% of the Firmicutes have AI-2 encoding gene *luxS* in their genome,^106^ which also implies that the composition of the microbiota could be controlled by QS. Interference of the QS system of the pathogen through analogues of QS signals is considered an elegant approach to curb the process of pathogenesis.^138^ Native gut microbiota *Blautia obeum* effectively inhibits the colonization of *V. cholerae* through the production of AI-2 synthase (*luxS*),^126^ whereas *V. cholerae* successfully establishes colonization in the absence of *B. obeum* (Figure 3b). It has been delineated that colonization of *V. cholerae* reduces when the mice received *E. coli* that harbor *luxS* of *B. obeum*. It has been further found that the expression of *luxS* of *B. obeum* is correlated with the restriction of *V. cholerae* possibly through VqmA-mediated novel pathway (Figure 3c). Since AI-2 has been widely produced by different gut bacteria, it could shape the community composition after antibiotic treatment (Figure 2a). AI-2 has been found to favor the colonization of Firmicutes, specifically a group of AI-2 producing bacteria.^106^ Thus, it can be concluded that QS plays a role in restoring the bacterial community.

It was found that LuxS of EHEC is also involved in the production of previously uncharacterized autoinducer-3 QS signal.^139^ Though the human gut consists of diverse bacteria capable of producing acyl-homoserine lactone (AHL), classical AHLs have not been detected in the gut environment.^140^ This might be due to the detection limitation of the sensor organism and lack of advanced technology for identifying AHLs from gut samples. Gut microbiota might harbor novel types of QS signals and receptor systems for interbacterial communication. In support of this, gut microbiota produces a novel type of AHL 3-oxo-C12:2, which is predominantly found in healthy individuals.^134^ The guts of patients with inflammatory bowel disease (IBD) have restricted biodiversity and reduction of Firmicutes diversity. During dysbiosis, such novel signal is reduced in the patient (0.25 ± 0.15 nmol/g of feces), whereas healthy individuals harbor abundant AHL (2.62 ± 0.80 nmol/g of feces). Despite Firmicutes (Erysipelotrichaceae, Ruminococcaceae, *Roseburia, Blautia*, Lachnospiraceae and *Faecalbacterium prausnitzii*) is positively correlated with an increased amount of 3-oxo-C12:2, classical LuxI/LuxR homologues are not detected in Firmicutes, suggesting that Firmicutes phylum harbor undiscovered novel QS regulatory genes. Applying appropriate methodology for the extraction of AHL from feces could provide an opportunity to detect all ranges of AHLs present in the gut ecosystem. The administration of AHLs in a murine model will allow us to identify the role of these AHLs in restructuring the gut microbial communities.^134^ Hence, the restoration of the bacterial community structure is possible with QS mechanisms. Exploring the correlation between bacterial diversity and QS signal repertoires could aid us to develop novel therapeutics for restoring bacterial diversity during dysbiosis.

Recently, it has been reported that probiotic *Bacillus* sp. capable of producing fengycin, a type of lipopeptide, restricts *Staphylococcus aureus* colonization through QS inhibition.^127^ Fengycin reduces Agr-mediated QS signaling in *S. aureus*. Perhaps, fengycin acts as an analog of the autoinducer peptide, which competitively binds with the respective receptor for inhibiting the QS mechanism of *S. aureus* (Figure 3d). Murine intestine colonized with *S. aureus* has effectively been eliminated by wild-type *Bacillus* than the *Bacillus* strain defective in fengycin (Figure 3e), implying that fengycin plays a key role in the restriction of *S. aureus* colonization. Several human-associated microbiota have been found to produce molecules that interfere with QS regulation of the pathogens and protect the host from infection.^141–143^

Apart from bacterial metabolites, the host has a multitude of molecules that directly influence the gut microbiota. Host cells produce several chemically diverse molecules, including serotonin, nitric oxide, autoinducer-2 mimic, epinephrine, ethanolamine and dynorphin, which could interfere with QS mechanisms of host-associated bacteria.^139,144–148^ Host-derived molecules either repress or activate the QS mechanisms of gut bacteria. For instance, nitric oxide inhibits the virulence factor production in *S. aureus* by nitrosylating the AgrA, thereby restricting the transition of commensal to a pathogen.^147^ In contrast, in response to host-derived asparagine Group A *Streptococcus* strain increases the production of QS signal SilCR, resulting in overproduction of bacteriocin.^149^ Consequently, bacteriocin-producing cells monopolize the niche by eliminating competitors. MicroRNAs are a non-coding short nucleotide sequence that inhibits the post-transcriptional mechanism by annealing with target mRNA. Intestinal epithelial cells derived microRNAs (miRNAs) modulate the gene expression of *E. coli* and *Fusobacterium nucleatum*.^150,151^ Hence, host miRNAs could manipulate the gut microbiome for the benefit of the host’s health.

## Impact of bacterial interactions on the structure of the gut microbial community

There is a strong correlation between the composition of gut microbiota and human health. Perturbation in the composition of the gut microbial community is always associated with several diseases, including obesity, cardiovascular disease, type 2 diabetes, and irritable bowel syndrome.^152^ Intrinsic interactions between bacteria precisely determine the composition of the community and increase the fitness of the host.^153^ Human gut microbiome is strongly conserved across hosts, but the taxonomic composition is diversified in each individual,^154^ thus confirming that the community function is not associated with specific microbial diversity. Though bacteria within the gut microbial community is organized depending on functional genes, factors involving in the selection and assembly of bacteria are not yet understood. We propose that molecular trafficking between bacteria might also play a pivotal role in the assembly of functional diversity. If we ask why spatial stratification and community assembly are essential for the function of the community, it might be due to the following reasons: i) to secure public goods from the surrounding exploiting cheater cells; ii) proximate localization of kin cells allows QS signals to bind with cognate receptors, eventually resulting in higher production of QS traits; iii) heterogeneous population of kin cells help each other for nutrient acquisition and iv) exporting essential commodities to sibling cells.

Microbiota gain survival fitness by acquiring different types of molecules from the neighboring cells through conduits or diffusion. The cross-talk between these machineries might be required to maintain microbial balance. Machineries such as CDI, T6SS, T7SS and nanotubes are likely to have similar attributes in terms of utilizing the common pool of EI pairs. Though each secretory system has a unique architecture, all systems deliver effectors that usually target conserved essential features of the competitors.^155^ While CDI and T6SS mediate interactions between gram-negative bacteria, T7SS contributes to the interaction between gram-positive bacteria. Besides, MVs, nanotubes and QS mechanisms mediate communication in both gram-positive and gram-negative bacteria. Now, the question arises as to how the EI reservoir is prevalent in both gram-positive and gram-negative bacteria. HGT plays an indispensable role for the existence of the EI resource in both bacterial systems. It is highly possible that nanotubes function as a bridge between genetically unrelated bacteria for sharing the molecular pool. Since T6SS and nanotubes mediate HGT, intrinsic crosstalk is likely between such machineries. Another example of the cross talk is that likely to occur between nanotubes and T6SS in *P. mirabilis*. For instance, discrimination of non-self cells occurs through the exchange of IdsD, where the transport is mediated by T6SS. It is frequently found that the inner diameter of the T6SS Hcp tube is ~40 Å.^156–158^ Though the export of IdsD is dependent on T6SS, how such bigger sized protein pass through the Hcp tube of T6SS is yet to be deciphered.^71^ Since the width of the nanotubes is shown to be more than 40 nm,^57^ *P. mirabilis* might be utilizing nanotubes for the exchange of IdsD, as a result of cross-talk between T6SS machinery and nanotubes. Hence, gaining knowledge of these critical bacterial interactions is the need of the hour.

## Conclusion and future directions

Based on the existing scientific evidence, both microbiota and pathogens possess multiple machineries, namely, CDI, T6SS, T7SS, nanotubes, MVs and QS, which function either in a contact-dependent or independent manner. In line with recent findings, it could be concluded that T6SS and QS mechanisms manipulate the human gut microbial composition. It was demonstrated that QS signals such as AI-2 and 3-oxo-C12:2 precisely restructure the composition of the Firmicutes community. While T6SS governs the composition of *Bacteroidales*, T7SS contributes in the structuring of Firmicutes diversity. Besides, CDI is likely to determine proteobacterial diversity. Despite CDI, T7SS, nanotubes and MVs have been shown to govern the local microbial community, the impact of these systems on the entire gut microbial community is not yet revealed. Therefore, an in-depth study on these secretory systems will usher in a new era in the field of human gut microbiota and provide ample opportunities to develop therapeutics in a new dimension. By understanding their survival strategies, potential pathogens could be selectively eliminated from the community. Since pathogens tend to survive in the host by producing virulence factors through the QS mechanism, it is, therefore, possible that targeted therapy could be achieved by targeting the communication system of the pathogens, which is key to their survival. In *P. aeruginosa*, both CDI and T6SS are regulated by RsmA regulator.^33,159^ Considering such a common regulator of secretory systems of the pathogens could be a potential therapeutic intervention to restrict pathogenesis. Many seminal research findings have highlighted that these machineries serve as a double-edged sword, having the potential to exhibit both co-operative and competitive behaviors. However, many key questions, like the following, are yet to be clearly answered. How does the CdiA-toxin domain get into the recipient cell? Does CDI-mediated aggregation induce QS of the microbiota in a highly dynamic environment, like gut? Does T7SS confer cooperative behaviors on microbiota? Does T7SS form a conduit for the translocation of toxins into the recipient cells? What are the receptor proteins required for establishing interaction through the T6SS?

Since gut microbes produce several previously undescribed molecules, utilization of such potential gut microbes for the restoration of microbial balance is a nuanced approach.^160^ Fecal microbial transplantation (FMT) is being used to restore microbial balance in the gut of IBD patients. FMT provides beneficial effects to the patients; however, imparting excess nutrients and amino acids could possibly favor the expansion of pathogens.^161^ Therefore, the donor’s microbial population has to be metabolically analyzed before FMT. Though antibiotic treatment appears to be effective for microbial restoration in IBD patients, the emergence of antimicrobial resistance (AMR) in pathogens poses a grave threat to global health. World Health Organization urges that AMR is an emerging threat to global health, food security and development. Dissemination of AMR genes carrying plasmids between pathogens^162^ allows them to thrive in a complex environment, like gut. Traditionally, type IV secretion system (T4SS) has been viewed as a machinery that can facilitate the spread of AMR genes. The presence of T4SS machinery encoding genes on conjugative plasmid^163^ suggests that pathogens recruit T4SS for conjugation. Recently, it was found that bacteria utilize machineries such as nanotubes, T6SS and MVs for spreading adaptive resistance genes,^40,91,^^164^ thus contributing to the emergence of multidrug-resistance (MDR) super-bugs. We believe that the manipulation of bacterial secretory machineries will allow us to fight against AMR. Identifying the proteins and metabolites responsible for inter-bacterial interactions paves the way to identify target pathogens and to develop novel methods to prevent the spread of AMR genes through HGT. Hence, venturing on novel therapies will enable us to restrict the overexploitation of antibiotics. To summarize, inter-bacterial interactions in the polymicrobial community largely influence the health status of the host. Hence, exploring the correlation between inter-bacterial molecular trafficking mechanisms and gut microbiome’s structure and function will lead to a holistic understanding as well as future medical solutions in this domain.

## Supplementary Material

Supplemental Material

## Abbreviations

Contact-dependent inhibition CDI

Contact-dependent signaling CDS

Type VI secretion system T6SS

Quorum sensing QS

Membrane vesicles MVs

Type V secretion system T5SS

Mobile genetic elements MGEs

Horizontal gene transfer HGT

Type VII secretion system T7SS

Type III secretion system T3SS

Integrative conjugative elements ICE

Genetic architecture GA

Acquired interbacterial defence AID

Pseudomonas quinolone signal PQS

Bacteroides ovatus BO

Bacteroides vulgatus BV

Autoinducer-2 AI-2

Fecal microbial transplantation FMT

effectors-immunity EI

Enterohemorrhagic *E. coli*EHEC

Acyl homoserine lactone AHL

Inflammatory bowel disease IBD

microRNA miRNA

Antimicrobial resistance AMR

Multidrug-resistance MDR
