# Supplementary material for: Molecular trafficking between bacteria determines the shape of gut microbial community
Source: Gut Microbes. 2021 Aug 30;13(1):1959841. doi: 10.1080/19490976.2021.1959841 (PMC8432619; doi:10.1080/19490976.2021.1959841)

**Supplementary Fig.1.** Intrinsic interactions between different set of mechanisms with quorum sensing system. CDI, T6SS and membrane vesicles systems are regulated by QS machinery in certain bacteria.[^97^](#_ENREF_97)^,^ [^162^](#_ENREF_162)^,^ [^163^](#_ENREF_163) Reciprocally, CDI, T6SS and nanotubes mediated interactions keep the cells in the close proximity, which enables them to communicate easily through QS signaling molecules in the given niche.


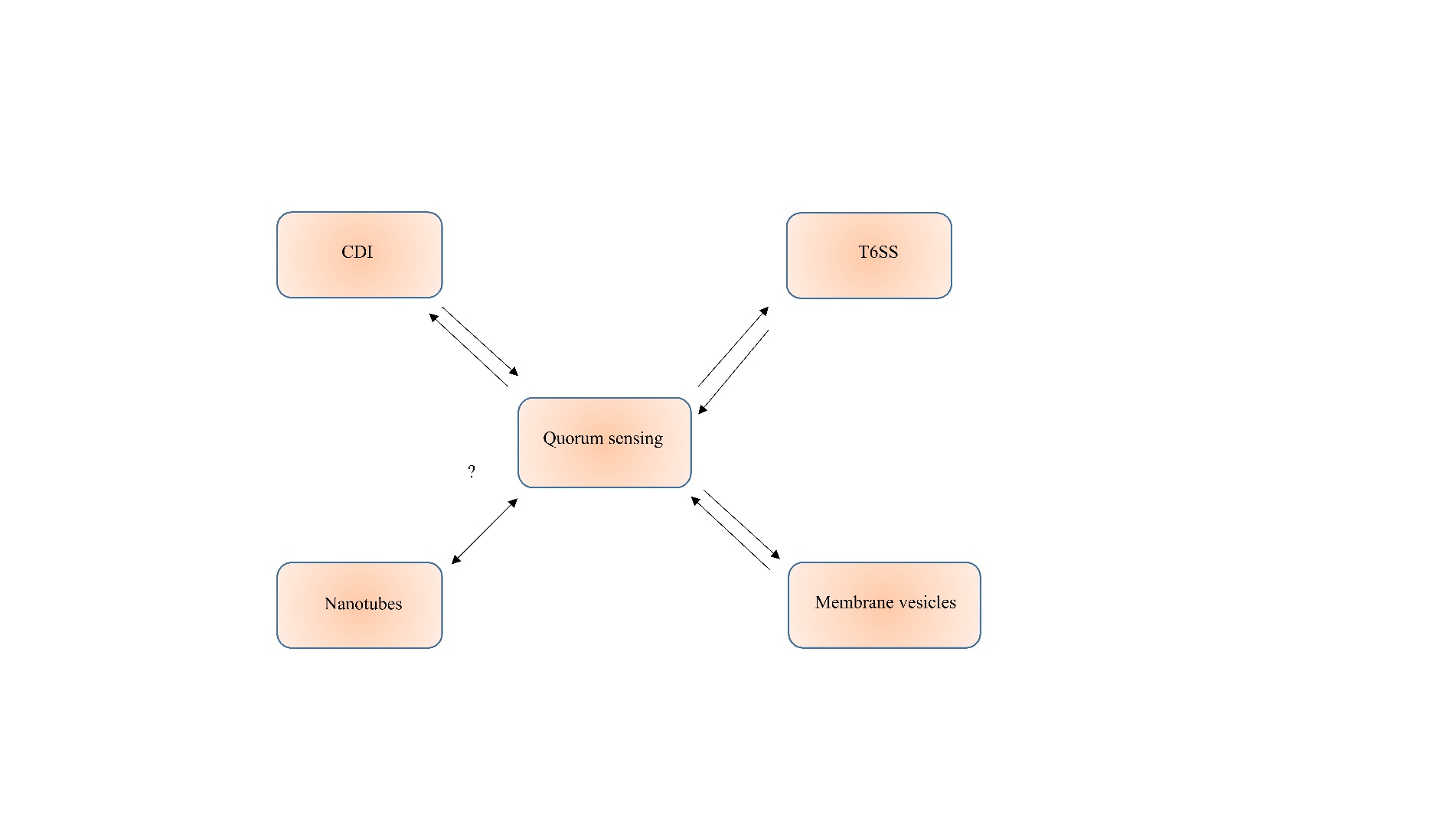

Supplement: Supplemental Material [file KGMI_A_1959841_SM1036.docx]
